# Long-term cyclic use of a sample collector for toilet-based urine analysis

**DOI:** 10.1038/s41598-021-81842-z

**Published:** 2021-01-26

**Authors:** Mikail Temirel, Bekir Yenilmez, Savas Tasoglu

**Affiliations:** 1 Department of Biomedical Engineering, University of Connecticut, Storrs, CT 06269 USA; 2 Department of Mechanical Engineering, University of Connecticut, Storrs, CT 06269 USA; 3 Department of Mechanical Engineering, Koç University, 34450 Sariyer, Istanbul, Turkey; 4 Boğaziçi Institute of Biomedical Engineering, Boğaziçi University, 34684 Çengelköy, Istanbul, Turkey; 5 Koç University Arçelik Research Center for Creative Industries (KUAR), Koç University, 34450 Sariyer, Istanbul, Turkey; 6 Koç University Research Center for Translational Medicine, Koç University, 34450 Sariyer, Istanbul, Turkey

**Keywords:** Biotechnology, Assay systems

## Abstract

Urine analysis via a toilet-based device can enable continuous health monitoring, a transformation away from hospital-based care towards more proactive medicine. To enable reliable sample collection for a toilet-attached analyzer, here a novel sample collector is proposed. The applicability of the proposed sample collector is validated for long-term use. Geometric parameters of the 3D-printed sample collector are optimized. The collected and leftover volumes are quantified for a range of urination speeds and design parameters. For long-term cyclic use, the protein concentrations of samples are quantified and the effectiveness of washing the sample collector is assessed.

## Introduction

A revolution of the healthcare industry is imminent: a transformation from reactive, hospital-center care to a proactive, person-centered approach^1^. Such a change requires a commensurate initiative to develop and implement affordable, easy-to-use technologies capable of continuous health monitoring. Routine health monitoring enables longitudinal tracking of personal health criteria, incorporating diagnostic capabilities into everyday life, as opposed to necessitating a visit to the doctor^2^. When one visits the doctor, it is common practice to perform a blood test via an intravenously collected blood sample. Blood tests have also been adapted for at-home use, as exemplified by handheld glucose monitors, which still require a painful finger prick to collect a small quantity of blood. Whether at the doctor’s office or in one’s home, blood is not suitable for routine monitoring due to the invasive collection process. In an effort to develop non-invasive health monitoring technologies, considerable research and development efforts have focused on wearable sensors^3^. As only a limited amount of information can be obtained from heart rate and dissolved oxygen measurements from the surface of the skin, there is a growing interest in sweat analysis for health monitoring via wearable sensors^4–15^. Nevertheless, there are several barriers to the successful implementation of sweat analysis. One of the most significant limitations is the minute volume of sweat excreted per unit time, per unit area of skin, compounded with the effect of accumulated sweat^16^. Various methods can be used to locally stimulate sweat release. However, existing methods are only intended for a single use and are not suitable for prolonged use^17–21^. Despite the ubiquitous use of health trackers (such as smart watches/bracelets or smartphone-based applications) and recent developments of sweat analysis tools, physical-activity data and sweat analysis have certain limitations for providing meaningful or continuous health data.

**Figure 1:**
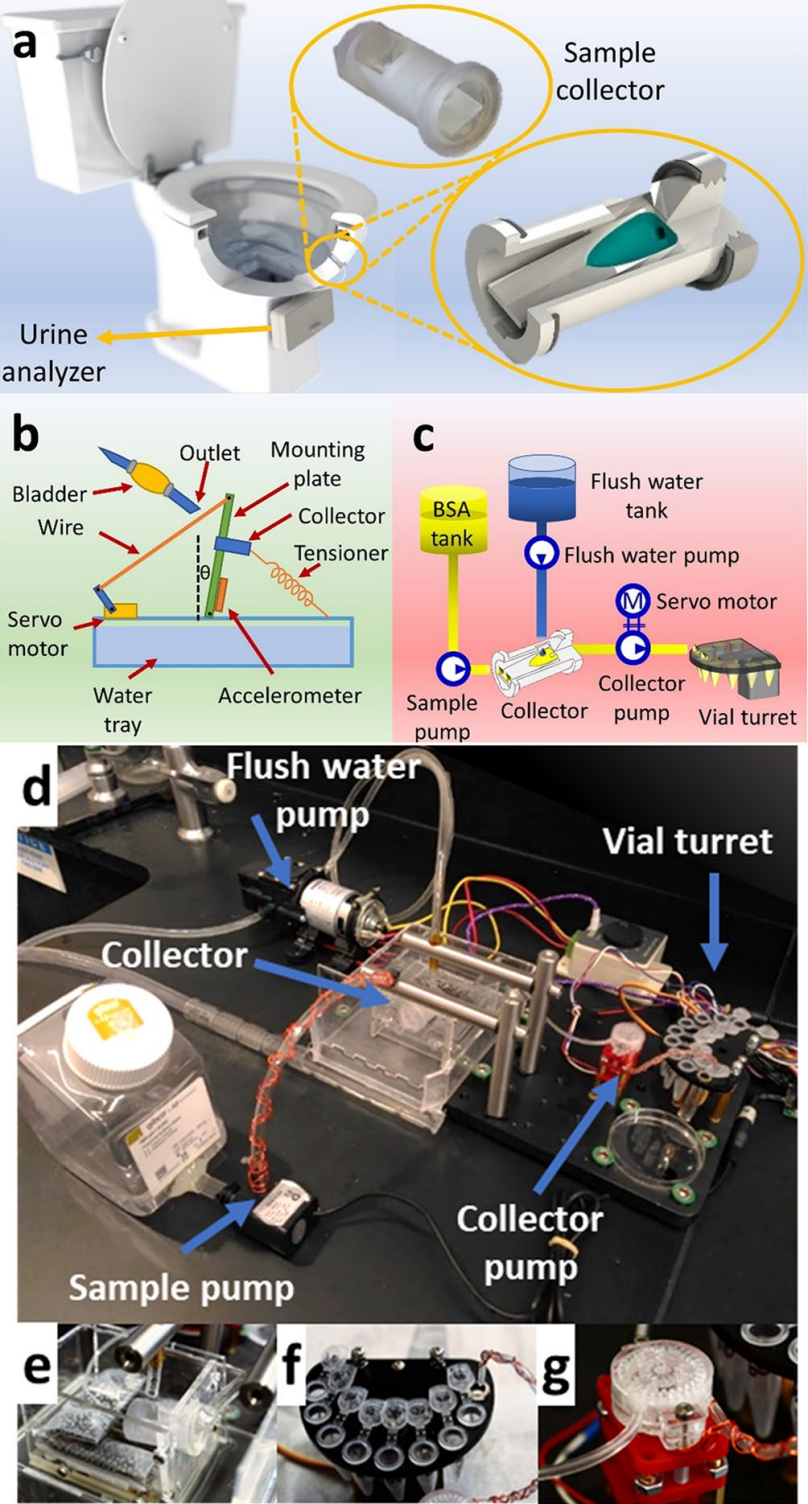
Design and characterization of a few hundred microliter-scale sample collector for a toilet-based urine analysis module. (**a**) Illustration of a sample collector and a non-disclosed urine analyzer integrated into a toilet. The insets show the 3D-printed prototype and further CAD details of the reservoir where the liquid will be collected. (**b**) Schematic view of the experimental setup for optimizing the collector design, composed of an acrylic plate to hold the collector, an accelerometer to measure and two servo motors to control the angle of the plate, a pump, and a 10 mL falcon tube with a drill hole at the bottom to act as an ejector. (**c**) Hydraulic circuit of the experimental setup for assessing the long-term cyclic use of the collector, including the flush water pump, sample pump, collector pump, collector, and vial turret. (**d**) Image of full experimental setup for studying the long-term cyclic use of the sample collector. (**e**) The collector held at a 10° angle by a laser-cut acrylic holder, positioned in a plastic tray. (**f**) Vial turret containing 8 vials and one drainage tube. The turret actuated by an attached servo motor that rotates 20° clockwise for each collection. (**g**) 3D-printed custom-designed peristaltic collector pump powered by a DC motor located under the pump, held in place by a laser-cut acrylic holder.

Another biofluid traditionally used by doctors is urine; the majority of people have experienced providing a urine sample in a cup for tests^22^. However, urine tests for personal use have traditionally been limited to single-use, single-purpose tests, such as pregnancy, ovulation, or drug tests^22^. While continuous blood monitoring via implantable devices is a distance away in the future and current efforts are considering sweat for continuous monitoring, a reinvigorated focus on urine for health monitoring purposes is well warranted for the wide array of analytes it possesses, in addition to the growing list of biomarkers and proteomics^23–26^. Urine serves as a more promising sample for ubiquitous health monitoring because unlike blood, urine is easy to non-invasively collect, and unlike sweat, urine is easy to collect in large volumes^22,23,27,28^. Urine proteomics has facilitated rapid and comprehensive urinary biomarker discovery^24,27^, revealing an abundance of urine-based biomarkers for specific conditions, particularly cancer and kidney-related issues^2^. In fact, the components found in urine are a direct result of glomerular filtration, tubular reabsorption, and secretion; therefore, the abundance of informative biomarkers present in urine reflects that in blood but with a lower level of complexity^1^. Another advantage of using urine is that it is completely non-invasive. While sweat monitoring is also non-invasive, it requires direct contact with the skin; only urine collection is totally free of contact with the body and, thus, is the most likely to be compatible with the behavioral preferences of the average user. Urine collection is painless, non-invasive, and does not cause any physical discomfort. Furthermore, to make urine collection discrete, burden-free, and able to be done in the privacy of one’s own restroom, we propose to develop a urine sample collector that can be mounted to a regular toilet bowl. The concept here (Fig. 1a) is to design a sample collector capable of collecting small volumes of urine; the collector can then direct the collected sample towards a custom toilet-based urine analysis module, thereby providing point-of-care urine analysis testing—the whole process occurring seamlessly in the background while providing facile and private collection and monitoring. We experimentally optimize the design parameters, such as the collector shape, drain angle, and mounting angle, to ensure that the amount of collected sample is consistent and that leftover sample is fully discarded after flushing. With practicality in mind, the sample collector must be robust for long-term cyclic use. For long-term use, a variety of reliability concerns must be considered, including consistency in volume collection, fouling by protein cross-contamination, and effectiveness of the cleaning/flushing between collections.

Herein, a novel platform is demonstrated for which any toilet-based urine sample collector design can be tested. Furthermore, the results presented in this paper are independent of the outer form factor of the sample collector design. Additional collector designs can be tested with this novel automated platform, which provides sample handling, collection, quantification, and flushing capabilities. Using this setup, the applicability of the designed sample collector is validated for long-term cyclic use by quantifying the collected volume and protein concentration (via plate reader and commercially-available dipsticks) of collected samples for 100 cycles according to these aforementioned considerations. Water is shown to be a sufficiently effective flushing liquid for cleaning the proposed sample collector. Since the flushing liquid of all toilets is water, the collector is deemed suitable for toilet-based applications. We envision that this design will enable facile and user-friendly urine sample collection for regular health monitoring. We hypothesize that frequent data collection and longitudinal processing will provide general data over time, though not necessarily in real time at this point; the enormous amount of data collected can be used to identify disease trends and predictors. Our work here represents the early stages of a novel technology concept to enable the future of healthcare with routine health monitoring by processing urine via seamless integration into users’ daily routine, with no expectations of behavioral change.

## Materials and methods

### 3D printing of sample collector

Three different 3D printers were used to fabricate the prototype sample collectors: (1) A stereolithography-based 3D printer (Form 2) with clear resin (RS-F2-GPCL-04, FormLabs Inc, Somerville, MA, USA), (2) an inkjet 3D printer (Objet30 Prime) with RGD-720 resin (Prairie, MN, USA), and (3) a fused-deposition-modeling 3D printer (Makerbot Replicator) with poly(lactic acid) filament (PLA, Makerbot, New York City, NY, USA). The final custom-designed collector device was fabricated with the FormLabs Form 2 3D printer with clear resin (RS-F2-GPCL-04, FormLabs Inc., Somerville, MA, USA) using the highest resolution possible with the Form 2 (0.025 mm) with the default support settings (density = 1, point size = 0.6 mm). Further details regarding the 3D printing of the sample collector is provided in the Supplementary Materials.

### Fabrication of experimental setups

For the characterization and optimization of the sample collectors, the experimental setup, shown schematically in Fig. 1b, consisted of the following components: a micro-servo motor (MG90S, Tower Pro, Taiwan), a three-axis accelerometer module (MMA7361, LC Studio, China), an Arduino Nano microcontroller (Arduino LLC, Somerville, MA, USA), a water pump (BYT-7A102, Bayite, China), a pulse-width-modulation (PWM) DC motor driver (Meimotor, Guangzhou, China), and a 3 mm cast acrylic sheet (McMaster-Carr, Princeton, NJ, USA). Solidworks 2017 3D design suite (Dassault Systémes, Vélizy-Villacoublay, France) was used for computer-aided design. FluorAcryl 6298 UV curable hydrophobic coating (Cytonix LLC, Beltsville, MD, USA) was used as a hydrophobic coating for the printed parts.

For studying the long-term cyclic use of the custom-designed sample collector, the following additional components were used in the mechanical design of the setup: submersible water pump (Mavel Star, 12V, China); micro-servo motor (MG90S, Tower Pro, Taiwan); water pump (BYT-7A102, Bayite, China); speed reduction DC motor with metal gearbox (Yosoo, 6V-60 RPM, China); Arduino Nano microcontroller (Arduino LLC, Somerville, MA, USA); a stepper motor driver controller (L293D, Texas Instrument, Dallas, TX, USA); two radial electrolytic capacitors (Nichicon2, 20 µF–16V and 10 µF–25V, Kyoto, Japan); 3 mm cast acrylic sheet (McMaster-Carr, Princeton, NJ, USA); 150 mm × 300 mm × 13 mm Solid Aluminum Breadboard and four 12.7 mm diameter, 100 mm long stainless steel posts (SAB15 × 30-M; Base Lab Tools Inc., Stroudsburg, PA, USA). Solidworks 2017 (Dassault Systémes, Vélizy-Villacoublay, France) was used as the CAD designer software. Figure 1c shows the hydraulic circuit of this secondary experimental setup.

The secondary experimental setup for studying long-term cyclic use of the sample collector is photographed in Fig. 1d. To hold the collector at a 10° angle, a mounting plate was designed in SolidWorks and laser-cut with a laser cutter (VLS2.30 CO_2_ laser cutter; Universal Laser Systems, Inc., Scottsdale, AZ, USA). The platform (Fig. 1e) was fixed on a plastic tray to keep the water contained. A vial holder (Fig. 1f), alternatively referred to as a vial turret, was also designed with SolidWorks and laser-cut. The turret holds eight vials and one tube for draining uncollected liquid. A servo motor was attached to the bottom of the vial holder, which rotates the holder plate 20° clockwise for each sample collection. 3D-printed parts of peristaltic pump were assembled (Fig. 1g) and controlled via a speed reduction DC motor. This collector pump takes protein sample from the collector and transfers the sample to the turret through thermoplastic tubing (inner and outer diameters of 0.793 and 2.38 mm, respectively) (TygonS3, Fisher Scientific, Pittsburgh, PA, USA). These three components of setup (collector holder, collector pump, and turret) were secured to a breadboard. A submersible water pump was used as the sample pump to transfer protein sample from the sample container to the collector via polyurethane (PU) tubing (inner and outer diameters of 4 and 6 mm, respectively). The outlet tip of the tubing was mounted with two stainless steel posts, positioned in an inverted “L” shape. In order to clean the collector after protein sample collection by the collector pump, we used a flush water pump that draws water from the water reservoir and directs it toward the collector via PU tubing. The tip of this tubing was also held with stainless steel posts, similar to the sample tubing.

### Overview of experimental design

Urine sample collectors were designed to be integrated into the bowl wall. Design parameters includes the diameter and the two sloped angles of the design: the incline of the center collection platform with respect to the designed mounting angle of 10° is called the collection angle, which guides the sample into the pool at the back of the collector, and the decline of the collection platform is called the drain angle, which guides the excess sample back into the bowl after testing and helps remove water during the cleaning cycle. The diameters, designed capacity, collection and drain angles of various tested designs are tabulated in Table 1. A visual guide for these various parameters is provided in Fig. S1 (in Supplementary Materials).

**Table 1:** Parameters of the designed collectors.

Design iteration	D (mm)	θ_c_ (°)	θ_d_ (°)	V (µL)
A	14	10	20	100
B	20	10	25	630
C	20	20	32	800

Design diameter (D), collection angle (θ_c_), drain angle (θ_d_), and designed capacity volume (V) for each design iteration.

The performance of the collectors was measured based on the amount of sample collected for each mounting angle relative to the vertical plane (0° means the collector is parallel and the acrylic plate is perpendicular to the floor). A pump was used to deliver the sample at measured flowrates of 10, 15, and 20 mL/s. The outlet tubing of the pump was directed towards the collector at a distance of 2.5 cm and 45° to the floor. For each experiment, 50 mL of sample was pumped from the dispenser; then, the sample remaining inside the collector was weighted using a precision balance (for samples less than 100 mg) or a pocket scale (for larger samples). The experiments were repeated after coating the collectors with a hydrophobic coating. Additionally, for evaluating the sample collector design, the leftover volume of liquid after flushing was considered. Fresh water enters from the top opening when the toilet is flushed to clean the collector. Ideally, there should be no water remaining in the collector to avoid diluting the next sample that will be analyzed. To simulate this cleaning cycle, 200 mL of water was poured from the back of the collector; then, the amount of water remaining inside the collector was measured.

For assessing the long-term use of the sample collector, the secondary experimental setup (Fig. 1c) was utilized. First, the sample pump runs for a half-second to direct 4 mL of sample towards/into the collector. After a one-second delay, the turret rotates to the first vial position, which is equivalent to a 20° rotation of the servo motor. There are nine positions on the turret: the first position is a drainage tube at the 0° position of the servo motor, the following eight positions are for subsequent samples. At the same time as the turret rotates, the collector pump starts running for 200 s to draw the sample from the collector to the vials. Then, the flush pump runs for 2 s and directs 75 mL of water to the collector. When the flush water finishes pumping, the turret returns to the drain position. Then, the collector pump is run again to drain the water from collector to the turret’s drain tubing. This pumping cycle was repeated every 3 h, which is approximately the time required for complete drying of the collector. The three pumps used for this cycle, each driven by a DC motor or servo motor, accept external PWM signals. A PC sends commands to the microcontroller, which converts the commands to PWM signals, and then it sends those signals to the related motors. We ran the experiment cycles, 3 h per cycle, via a MATLAB script by setting the number of cycles before commencement of the experiment.

Protein sample was prepared with albumin from bovine skin (BSA, Sigma-Aldrich, Louis, MO, USA) in PBS solution, at concentrations of 0.1, 0.3, 0.5, 1, 2, and 5 g/L, in a sterile hood. We have chosen this range of concentrations to augment the contamination seen by collector, although the protein concentration found in normal urine averages around 0.1 g/L. Once all BSA-in-PBS samples have been collected, we measured the weight of the vials and quantified the protein concentration with a Synergy H1 plate reader (BioTek Instruments, Inc. Winooski, VT, USA). Absorbance was selected as a detection method using the default read type and optic type (read type was endpoint/kinetic and optic type was monochromators) with 280 wavelengths.

## Results

### Design of sample collector

The objective was to collect undiluted urine samples so the collector was designed to be mounted above the water level of the toilet, as compared to collecting samples from the main bowl of the toilet. The urine sample collector was designed as a reservoir that is easy to target while a person is urinating. It has a cylindrical exterior shape in order to allow easy drilling and mounting into the sidewall of existing toilet bowls (Fig. 1a). The design presented here ideally collect urine samples between 100 and 500 µl. The current design is compatible with siphon-jet-flush toilet bowls and can be easily modified to fit into other toilet bowl types, such as gravity-fed-flush toilet bowls or urinals.

**Figure 2:**
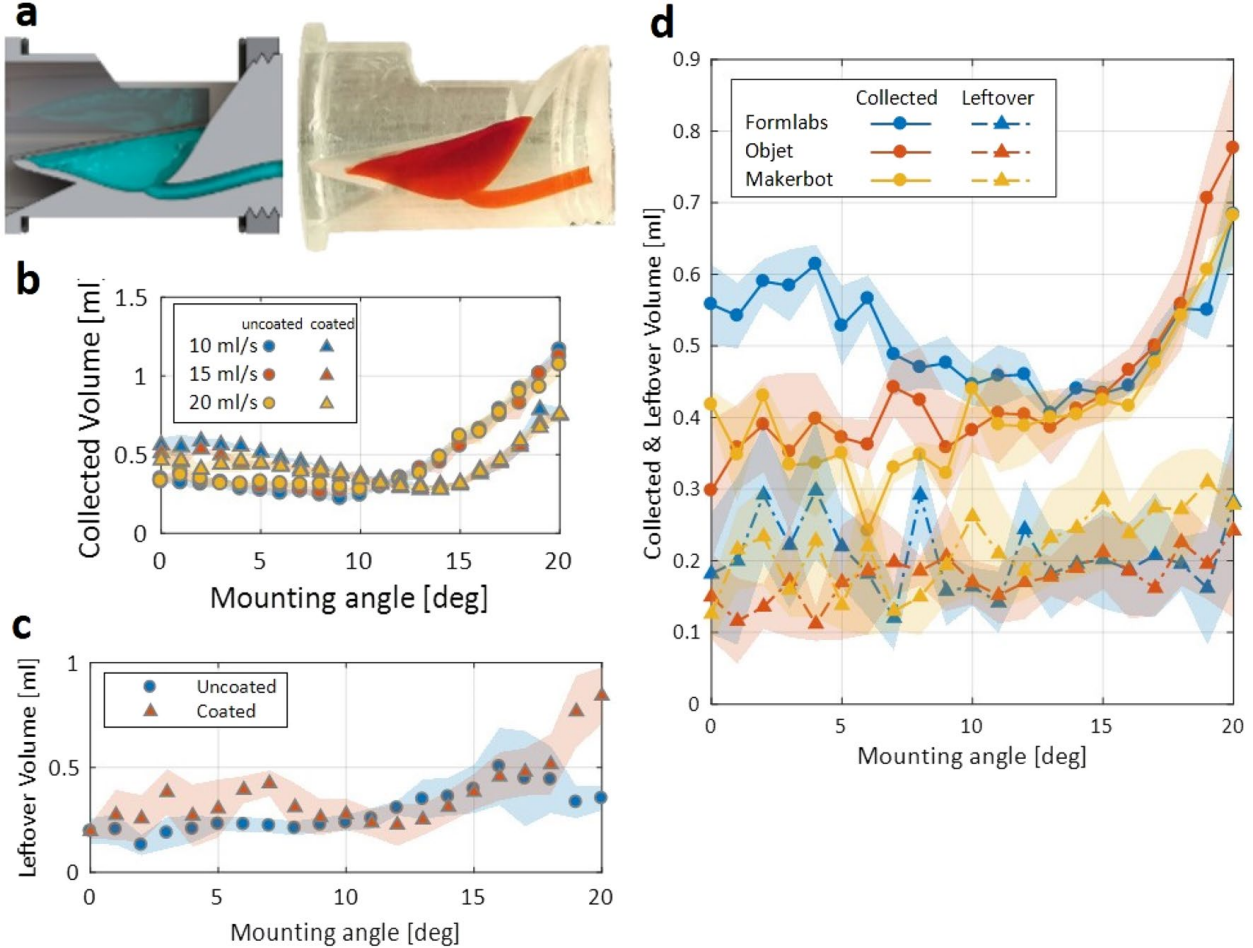
Characterization of the collected sample volume and leftover volume for the collector design. (**a**) Cross-sectional view of the collector design is provided (computer model, left, and photograph, right) (**b**) The amount of sample collected (mL) versus mounting angle (°) (n = 5). (**c**) The amount of leftover liquid after flushing. (**d**) The collected and leftover sample volumes for the collector fabricated by three different printers (Formlabs Form 2, Objet30 Prime, and Makerbot Replicator).

First, the volume of water collected by the sample collector was quantified. Cross-sectional views of the collector are shown in Fig. 2a. The results are shown in Fig. 2b. The data shows that the sample flow rate does not significantly affect the amount of sample collected. At low flow rates, the sample fills the collector gradually, while at high flow rates the momentum of the sample pushes extra fluid out of the collector. Second, the leftover volume after flushing the sample collector was quantified. The results are shown in Fig. 2c. It is seen that the amount of leftover water increases with the mounting angle. The collected volume and leftover volume results for other design iterations are shown in Fig. S2 (in Supplementary Materials).

To compare different printing technologies and materials, the sample collector was fabricated with three different 3D printers. In addition to stereolithography-based FormLabs Form 2 that was used for all previous experiments, inkjet-based Objet30 Prime and fused-deposition-based Makerbot Replicator were used to fabricate the design. The hydrophobic coating was not applied to facilitate better visualization of the surface finish and material. The experiments described above were repeated with a constant flow rate of 15 mL/s; the results are shown in Fig. 2d. The collectors fabricated with the Objet30 and Makerbot printers collected less sample at the relatively low mounting angles: at mounting angles of less than 7°, the collector printed by the FormLabs Form 2 held 0.5–0.6 mL of sample while the others held 0.3–0.4 mL. However, all collectors held similar amounts of sample at mounting angles above 13°. The printer used did not appear to influence the amount of leftover sample: all prints retained 0.1–0.3 mL of sample at all mounting angles. The printing performance results of various design iterations using the three different 3D printers are provided in Supplementary Materials, and are summarized in Fig. S3 (in Supplementary Materials).

### Protein concentration of collected samples

**Figure 3:**
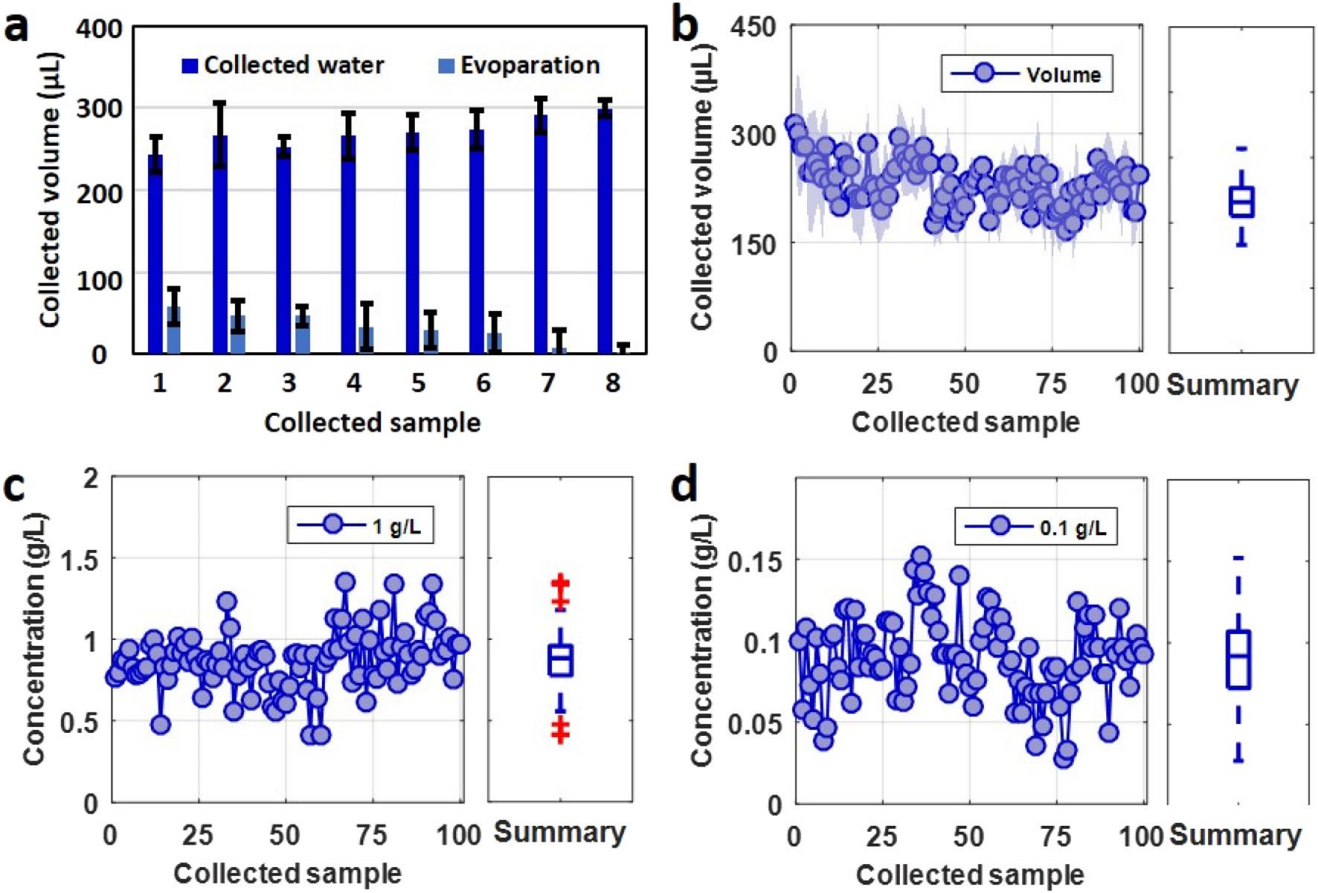
Cyclic sample collection and protein quantification for clean and pre-treated collectors. (**a**) Collected water volume and its evaporation rate in the vials of rotating turret for each cycle. The vial turret automatically collects a sample at 3-h intervals. Error bars show the standard deviation (n = 5). (**b**) Volume of samples collected by the custom-designed sample collector for each cycle. Total number of cycles, interval period per cycle, and total experiment time are 100 cycles, 3 h, and 300 h, respectively. The collected volume is summarized by a boxplot, at right. (**c**) Quantification of protein concentration in 100 collected samples via a plate reader for applied concentration of 1 g/L. Results show an average of approximately 0.87 g/L. The protein concentration is summarized by a box chart, at right. (**d**) Quantification of protein concentration in 100 collected samples via a plate reader for applied concentration of 0.1 g/L. Results show an average of approximately 0.09 g/L. The protein concentration is summarized by a box chart, at right.

To assess the volume and protein concentration of collected samples, the following cycle was performed: (1) Pump sample into the collector, (2) Collect sample into a vial, (3) Flush the collector with water, (4) Drain the collector, (5) Wait 3 h for the collector to dry. Figure 3a shows the volume of collected water for each cycle and the volume of evaporated water for each interval, indicative of the collecting capability of the collector. Water was collected for 8 trials, with 3-h intervals between each collection. At the end of the 8 trials, we closed the lid of vials of collected water and weighted them to determine the exact collected volumes. The vials collected at the beginning of experiment exhibited volume loss due to water evaporation since they remained open for a longer period of time, as compared to the last collected samples. The average volume of the collected sample is 270 µL. The volume of evaporated water was calculated by taking the last vial as a reference since it was closed immediately after collection. The rate of evaporation was approximately %3 per 3-h interval. Figure 3b presents the volume of collected BSA samples for each cycle. The total number of cycles, interval period per cycle, and total experiment time are 100 cycles, 3 h, and 300 h, respectively. The average collected volume and standard deviation are 250 µL and 66 µL, respectively, during the 100 cycles. A possible reason for the slight fluctuations is the evaporation of liquid in the vials after collection is complete. Due to the 24-h total experiment time, vials collected at night were not immediately closed after sample collection, compared to vials collected during the day which were closed in a timelier manner. Nonetheless, despite the relatively large range in the collected volume (plus/minus approximately 150 µL), the collected volume of all samples, even at the lower end of the range, is sufficient to analyze the collected sample via a plate reader, in addition to analyzing using a dipstick. The collected volume results are summarized by a boxplot, as seen the right-hand side of Fig. 3b.

Figure 3c shows the results of protein concentration in the 100 collected samples for an applied sample concentration of 1 g/L; quantification is performed using a plate reader. Even though the concentration of the applied sample is 1 g/L, the average concentration of the collected sample is 0.87 g/L. The surface of collector retains a very small amount of protein due to its surface roughness. The protein in the applied solution is able to stick to the collector’s surface during the sample pumping; as a result, when the sample is subsequently collected after passing through the collector, some of the protein adheres to the collector’s surface rather than being pumped into the vials, which causes the approximate 10% decrease in the concentration of protein in the collected samples, as compared to the concentration of applied protein sample. The protein concentration of the collected sample is relatively consistent with repeated cycles, indicated by an average standard deviation of 0.018 g/L for 100 cycles. The boxplot on the right-hand side of Fig. 3c summarizes the concentration results. To further demonstrate the applicability of the collector, the experiment of Fig. 3c was repeated for an applied sample concentration of 0.1 g/L. In this case, the average concentration of the collected sample is approximately 0.09 g/L, which is consistent with the results seen in Fig. 3c, showing a similar 10% decrease in protein concentration. The boxplot on the right-hand side of Fig. 3d summarizes the concentration results.

The range in the measurement of protein concentration should also be noted: as can be seen in the boxplot of 3c and 3d, the range is approximately plus/minus 0.5 g/L and plus/minus 0.05 g/L, respectively, or around 50% of the applied concentration. Although a single measurement in isolation may fall within this range, and therefore result in an inaccurate measurement, as samples are collected and measured over time, the results will create a stable trend localized around the average concentration. By focusing on trends over time, as opposed to individual measurements, these results can still prove useful in detecting changes based on trends. Furthermore, even in consideration of the relatively large range, the measurement is still specific enough to differentiate between healthy, moderate proteinuria, and extreme proteinuria cases.

### Validation using commercial dipsticks

**Figure 4:**
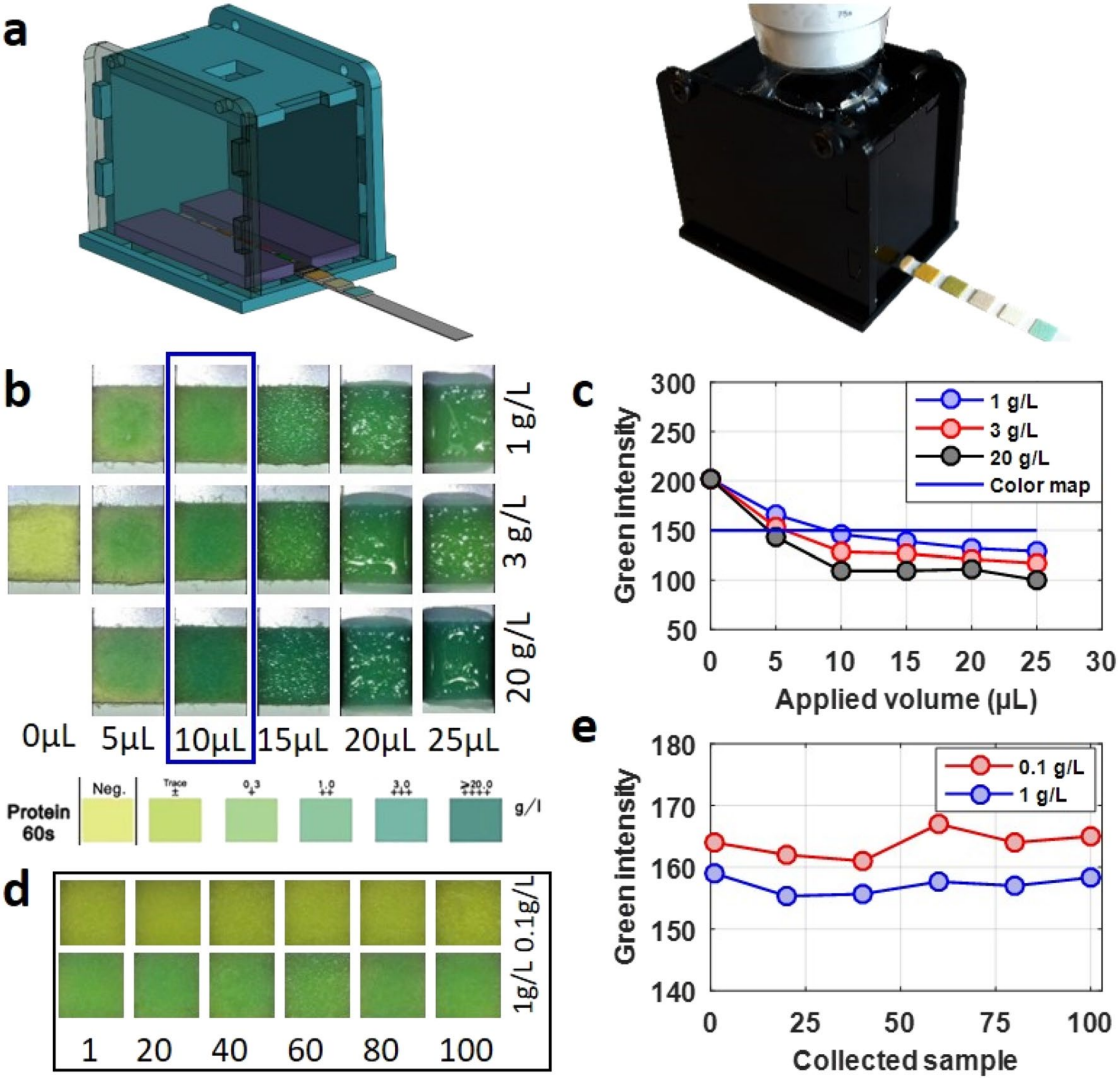
Characterization and validation of the setup with dipstick. (**a**) Imaging box to block outside light to capture an image of an inserted dipstick. Left: CAD drawing. Right: Assembled fabricated from laser-cut acrylic. (**b**) Optimization of the volume of liquid applied to the reagent strip. The results with different sample volumes are compared with the tabulated color map provided by the dipstick manufacturer. (**c**) Quantitative intensity analysis of the results shown in (**b**). (**d**) Strip color upon application of 10 µL of sample collected after running for one-hundred 3-h intervals. (**e**) Quantitative intensity analysis of the results shown in (**d**).

In order to quantify the protein concentration of the collected sample and validate the concentration read by the plate reader, we used commercially-available urine analysis dipsticks (Urinalysis reagent strip, HealthWiser LLC., CA, USA). Dipsticks function on the colorimetric detection method, a commonly used qualitative detection method, in which the color of the detection pad changes color in response to analyte concentration^29,30^. By imaging the detection pad, a quantitative concentration measurement can be obtained. We designed an imaging box to block outside light while capturing an image of an inserted dipstick, as shown in Fig. 4a, via a digital USB microscope (Carson eFlex 75 ×/300 ×, Carson, China). A CAD drawing of the designed box is seen on the left, while the right image shows the box fabricated from laser-cut acrylic. To ensure that the imaging box provided consistent images, we imaged two standard color cards which correspond to the color range of the protein colorimetric assay: yellow (Benjamin more, bright yellow, 2022-30) and green (Benjamin more, Fresh Lime, 2032-30). A 5 × 5 mm piece of each color card was cut and placed into imaging box, in the same position and orientation as a dipstick. 15 images of each of the two colors were taken in 1-min intervals. The green intensity at the center of the image of a 150 × 150-pixel square was measured with MATLAB (version R2016a). The result, as seen in Fig. S4 (in Supplementary Materials), shows the consistency of the measurement. Figure 4b depicts the results from optimizing the volume of sample applied to the dipstick for three different BSA concentrations (1 g/L, 3 g/L, and 20 g/L) which are concentrations tabulated by the color map provided by the dipstick manufacturer. Above, images are shown of different volumes of BSA samples, ranging from 0 to 25 µL, applied to the reagent strip to find the appropriate volume. Images were taken 60 s after sample was applied, as dictated by the dipstick manufacturer’s specifications. Captured images were analyzed in terms of green-channel light intensity via MATLAB in order to quantify the results. These results were compared with the tabulated color map provided by the dipstick manufacturer on the box. It is possible to see the color differences due to varying volumes and concentrations with the naked eye. For all concentrations, the color is noticeably darker after applying 10 µL. For volumes greater than 10 µL, the reagent strip does not absorb the whole applied volume. For quantitative assessment of the appropriate sample volume to be applied to the reagent strip, the portion of the color map corresponding to 1 g/L was cut and imaged in the imaging box; the green intensity of this color map square was similarly measured. Intensity results are plotted in Fig. 4c. Of the different volumes tested, the green intensity of a 10 µL sample applied to the reagent strip most closely matched the intensity of the 1 g/L color map; the selection of 10 µL was also verified by visual inspection. 10 µL of collected samples were applied to reagent strips, each sample collected after a set number of cycles (1, 20, 40, 60, 80, and 100). The dipstick results are shown in Fig. 4d. Quantitative analysis by green intensity of the results shown in Fig. 4d are shown in Fig. 4e. The intensity analysis demonstrates very consistent concentration results throughout the 100 cycles. Thus, the dipstick test validated the consistency of concentration measurement obtained via the plate reader. This experiment was repeated three times, represented by the standard deviation.

### Performance of cleaning the collector

Cleaning the collector and tubing is crucial for long-term use of the collector since remaining protein in the left-over flush liquid may have an effect on the next cycle. We have characterized the cleaning performance of the collector. Prior to the experiment, we made slight modifications to the MATLAB script to collect the left-over liquid after flushing, as opposed to collecting the protein sample. At the start of the experiment, 4 mL of BSA was pumped towards/into the collector. After introducing the sample, the collector pump drains the sample from the collector. Following the initial sample, 20 cycles, each of 10-min duration, were performed, where each cycle consisted of 2 s (75 mL) of flushing liquid directed at the collector, after which the collection pump ran for 200 s. The remaining time for each cycle was sufficient for drying, since when the flush liquid is collected, the collector is already wet from the collector being flushed after protein sample collection. This procedure was repeated for a range of protein concentrations from pure PBS up to 5 g/L of BSA: 0, 0.1, 0.3, 0.5, 1, 2, and 5 g/L.

**Figure 5:**
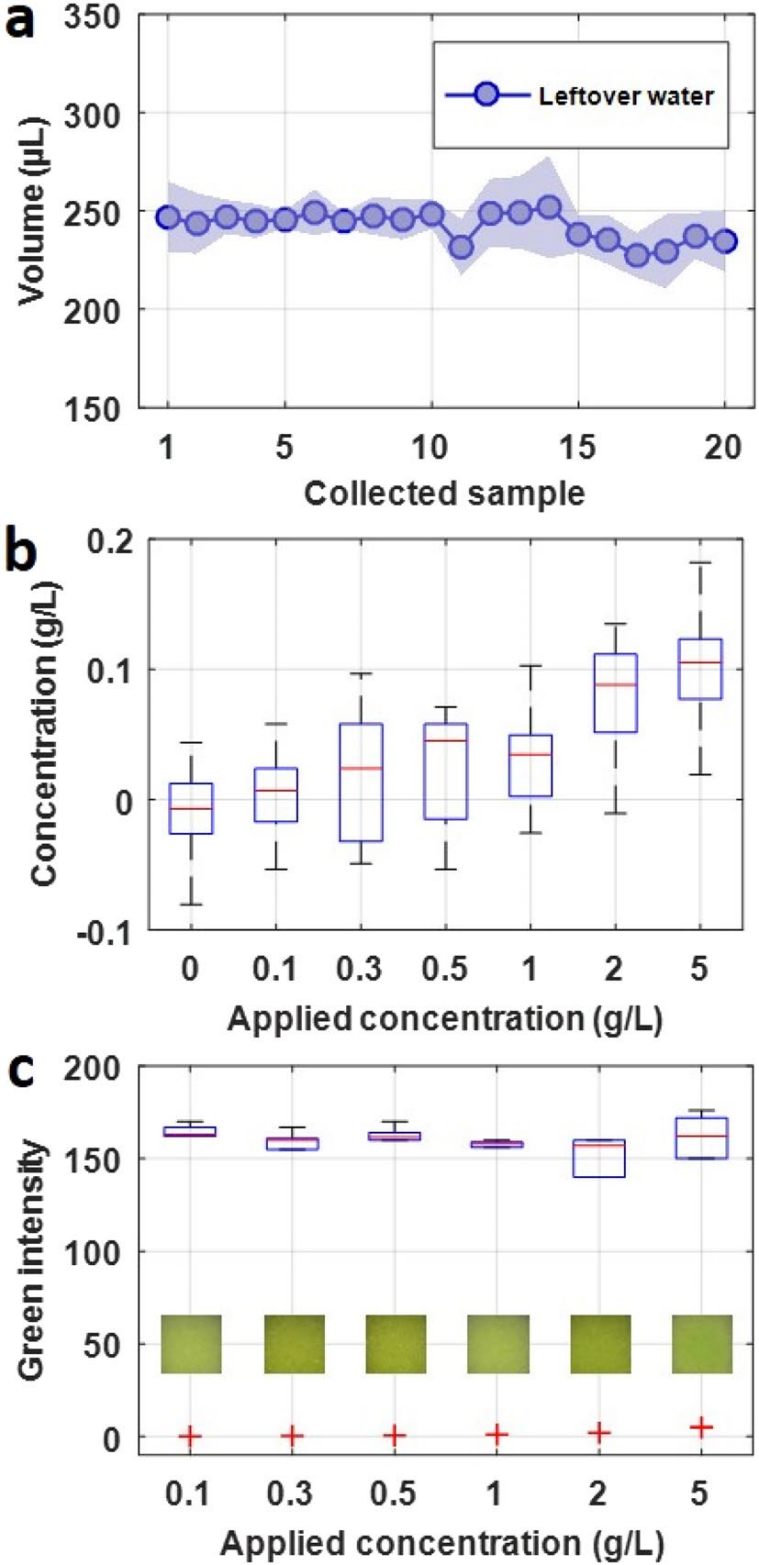
Performance of cleaning the collector and tubing. (**a**) Collected remaining/leftover volume using water as the flushing liquid for each cycle. Total number of cycles, interval period, and total time are 20 cycles, 10 min, and 200 min, respectively. (**b**) Quantification of protein concentration in 20 collected leftover samples via a plate reader for a range of applied concentrations. (**c**) Quantitative intensity analysis of dipstick protein assays. Each experiment is repeated 3 times, represented by standard deviation. Inset images depict the reagent strip color after application of 10 µL of collected leftover flushing liquid.

Figure 5a is the volume of collected left-over samples when using water as the flushing agent. Figure 5b shows the quantification, via a plate reader, of the remaining protein concentration for flushing liquid. Taking note of the 1 g/L applied concentration, the results showed a concentration in the leftover flushing water of approximately 0.035 g/L. This represents a reduction by over 95%. For all applied concentrations tested, this significant reduction in concentration in the leftover water is observed. Furthermore, it should be noted that for pure PBS (0 g/L), there was still an interquartile range of approximately ± 0.15 g/L due to the inherent noise in the plate reader measurement. This noise can also account for a portion of the range in measurements for all applied concentrations. A healthy individual may have a urinary protein concentration in the range of 0 to 0.2 g/L, so although the remaining protein concentration for higher applied concentrations is within this healthy range, the sensitivity of the device would not be affected for differentiating between healthy, moderate proteinuria, and extreme proteinuria cases. Furthermore, by increasing the volume of flushing liquid, further reduction in protein concentration may be achieved. Quantification of a dipstick protein assay is shown in Fig. 5c. Inset are representative images of the reagent strip on which 10 µL collected leftover water was applied. These results show that by a simple colorimetric test using commercial dipstick assays, the remaining protein concentration in the leftover water for all applied concentrations is relatively constant, meaning the device is sufficiently cleaned (i.e., no cross-contamination can be discerned using this measurement method).

## Conclusions

In this study, we have developed a urine sample collector that can be integrated into a toilet. It can ultimately be used to track the health of the user on a daily basis and, thus, facilitate the shift of healthcare from a reactive approach to a proactive one by detecting health problems before they become severe. In addition to optimizing the sample collector design, we applied a hydrophobic coating as a surface treatment and compared the results with those of uncoated counterparts. A custom experimental setup was fabricated, in which the mounting angle of the collector was varied and the volume of sample collected was measured with different sample flow rates. Moreover, a flushing cycle was simulated and the amount of water remaining in the collector was measured to ensure thorough flushing of the collector between uses.

The results show that the hydrophobic treatment does not have a significant impact on the volume of liquid collector. The amount of sample collected depends on the mounting angle: more sample is collected as the mounting angle increases. It was also found that the drain angle in the does not influence the amount of liquid remaining after cleaning as long as it is steeper than the mounting angle. Furthermore, three different 3D printers were tested for fabricating the sample collector. The results showed that the surface texture affects the collected volume when the mounting angle is close to horizontal: the rougher surfaces generated by the Objet30 and Makerbot 3D printers held less sample. However, the remaining water after flushing was similar in all the prints. Thus, it can be concluded that the proposed design holds a specified amount of liquid and can be cleaned during the flushing cycle. This design provides about 20° of tolerance for mounting in the toilet bowl, making it possible to collect and analyze urine unobtrusively for daily health tracking in almost any home toilet.

To validate the proposed toilet-based sample collector for long-term use, we designed a secondary experimental setup that collects samples for long durations by itself without any user intervention. The 3D-printed collector was mounted at a 10° angle. BSA sample (1 g/L) was collected for 100 cycles with 3-h interval. The volume and protein concentration of collected samples was measured as function of the number of the samples. Moreover, cleaning the collector was performed with two different flushing liquids by measuring the protein concentration of leftover liquids and the amount of remaining liquid. The results show that the volume of collected sample was relatively consistent over the course of the 100 cycles, where the small fluctuations seen were caused by evaporation of liquid. Likewise, there was no significant difference in the protein concentration for the 100 cycles.

## Future perspective

Healthcare in today’s society revolves around the hospital; however, society stands to benefit greatly from a transformation away from this reactive approach and toward a more proactive methodology^1^. This transformation calls for the development and implementation of technologies to enable preventative, evidence-based, person-centered care. These technologies must be affordable, compact, and compatible with continuous health monitoring in large populations. There exists an abundance of technologies for blood analysis. However, these tools are unlikely to lend themselves well to the continuous health monitoring of the future as they rely on sample collection, preparation, and analysis and are limited in their ability to be portable, affordable, and continuous^2^. Moreover, even the devices that are simple enough to be used at home (for example, glucose-monitoring devices) are unlikely to be widely implemented for generalized health monitoring because of the pain associated with obtaining blood samples. Recently, wearable sensing technologies have been developed and adopted for non-invasive continuous health monitoring; their widespread adoption has initiated this long-needed shift to person-centered medicine^3^. As only a limited amount of information can be obtained from heart rate and dissolved oxygen measurements from the surface of the skin, there is a growing interest in sweat collection and analysis for health monitoring via wearable sensors. However, there remain notable barriers against the implementation of sweat-based technologies. For example, the quantity of sweat collected per unit time is very minimal; a millimeter-scale sensor only receives several nanoliters of sweat per minute. Urine serves as a more promising sample for ubiquitous health monitoring due to the ease of collecting large sample volumes^22,27,28^.

Urine collection is non-invasive and simple since everyone urinates, with or without monitoring. We envision that urine analysis has great potential to be integrated into the everyday lives of the general population. Our proposed sample collector design will enable facile and user-friendly urine sample collection for regular health monitoring. We propose this urine sample collector as a means for reducing the manual steps required for routine urine monitoring. Additionally, by focusing on the larger picture of data trends, as opposed to individual measurements, the health of the user can be tracked over time. Furthermore, the device is low-cost enough to be replaced when necessary, for instance, after being exposed to abnormally elevated analyte concentrations. The aforementioned data trends can also be useful in indicating when such a replacement is necessary.

In future stages of the proposed collector, additional components would be designed to circulate and drain cleaning reagent (e.g. from an integrated reservoir) through the sample collector between uses^31,32^. This would help to reduce contamination over time. Additionally, there is a considerable amount of active research being reported for reducing biofouling and creating anti-fouling coatings^33^. Such coatings are a possible mediation strategy which could be pursued in future work. Furthermore, alternative materials which exhibit lower levels of biofouling may also be considered in future work. Lastly, with a focus on clinical relevance, the proposed device should be tested for a wide array of other analyte assays, in addition to protein concentration, in future studies and further research is required before this product can be used in a clinical setting. Further optimization of this device, in terms of usability in point-of-care and clinical settings, may include determining the recommended time period between replacement of the sample collector. As research in the field continues, the already wide range of analytes and urinary biomarkers will continue to grow. Simultaneously, the need for proactive healthcare continues to be ever-present. We see continuous health monitoring via urine analysis as the ideal application of this area of research for this great societal need.

## Supplementary Information

Supplementary Information.
