# Supplementary material for: Long-term cyclic use of a sample collector for toilet-based urine analysis
Source: Sci Rep. 2021 Jan 26;11:2170. doi: 10.1038/s41598-021-81842-z (PMC7838403; doi:10.1038/s41598-021-81842-z)
Supplement: Supplementary file 1 — Supplementary Information. [file 41598_2021_81842_MOESM1_ESM.pdf]

Long-term Cyclic Use of a Sample Collector for Toilet-Based Urine Analysis

Supplementary Materials

Mikail Temirel[1,], Bekir Yenilmez[2,*], and Savas Tasoglu[3,4,5,6,¥]*

[1]Department of Biomedical Engineering, University of Connecticut, Storrs, CT 06269.
[2]Department of Mechanical Engineering, University of Connecticut, Storrs, CT 06269.
[3]Department of Mechanical Engineering, Koç University, Sariyer, Istanbul, Turkey 34450.
[4]Boğaziçi Institute of Biomedical Engineering, Boğaziçi University, Çengelköy, Istanbul, Turkey 34684.
[5]Koç University Arçelik Research Center for Creative Industries (KUAR), Koç University, Sariyer, Istanbul, Turkey 34450.
[6]Koç University Research Center for Translational Medicine, Koç University, Sariyer, Istanbul, Turkey 34450.

*These authors equally contributed to this article.
¥Corresponding Author: Savas Tasoglu (stasoglu@ku.edu.tr)

Supplementary Materials and Methods

3D printing of sample collector and peristaltic pump

The Form 2 prints parts in a layer-by-layer manner onto a platform by scanning a laser through a pool of photo-sensitive resin in a specified pattern; the platform is moved vertically as the layers are cured. Thus, hollow shapes can be readily formed although supports may be necessary depending on the shape. The highest resolution possible with the Form 2 (0.025 mm) was used with the default support settings (density = 1, point size = 0.6 mm). All parts were cleaned in an isopropyl alcohol bath after printing to remove excess uncured resin; the support material was removed with a cutter and the surfaces were polished with sandpaper as needed. The Objet30 Prime prints parts from bottom to top using a row of piezoelectric nozzles. It ejects small droplets on the platform and cures them with an ultraviolet light source after each layer. Since droplets must be deposited onto a flat surface (i.e., they cannot be suspended in the air), a support material is necessary to fill hollow regions and can be cleaned out mechanically using a brush and pressurized water after the part is printed. No additional surface polishing is necessary with this printer. A glossy surface finish with "high-speed" setting (28 μm resolution) was used for all parts. In the fused-deposition printing via Makerbot Replicator, the filament is heated prior to extrusion and cooled on the platform to form a rigid part. Hollow shapes can be printed but supports of the same material (PLA) are used for hanging structures with sharp angles and must be removed mechanically after printing. A surgical knife was

used to remove these supporting structures and the surface was smoothed with sandpaper. The print quality was set to standard (0.20 mm layer height) with a 10% infill, two shell layers, and the raft and support options enabled. The final custom-designed collector device was fabricated with the FormLabs Form 2 3D printer with clear resin (RS-F2-GPCL-04, FormLabs Inc., Somerville, MA, USA). The Objet30 Prime 3D printer was used to print a custom-designed peristaltic pump, consisting of three geared rollers, one center roller, and bottom and top geared lids. The chosen resin was transparent resin RGD-720 (Prairie, MN, USA).

Fabrication of experimental setups

Characterization and optimization of sample collector

A mounting plate was designed in Solidworks to hold the collectors in place. The design was laser-cut from a 3mm-thick acrylic sheet with 100% power (30 W) and 4.3% speed in a single pass (VLS2.30 CO_2 laser cutter; Universal Laser Systems, Inc., Scottsdale, AZ, USA) and fixed over a plastic tray, which serves as a water reservoir representing the toilet bowl. The mounting plate was hinged at the bottom with two ball bearings to facilitate smooth movement. Two radio control (RC) servomotors were attached to the front of the reservoir and connected to the front of the mounting plate with wires, while an accelerometer was used to measure the angle of the plate. A rubber band was attached to the mounting plate at the back to hold it steady by applying tension against the wires. The pump draws water from the reservoir, pumps it via polyurethane (PU) tubing (inner and outer diameters of 4 and 6 mm, respectively) through a custom nitrile bladder near the outlet, and dispenses it toward the collector. The custom bladder is added immediately before the outlet in the tubing to dampen the periodic ebbs and flows caused by the reciprocating pump, thus generating a smoother and more anatomically correct flow. This pump is driven by a DC motor that was modified to accept external PWM signals. The microcontroller receives commands from a PC, sends a corresponding PWM signal to the pump, controls the servomotors to set the mounting angle, and measures the actual mounting angle using an accelerometer. A calibration routine was conducted each time the collector was changed to find the pulse width that needs to be sent to the servomotors to attain the required angle. To run an experiment, the user inputs the mounting angle, flow rate, and duration of the flow via a MATLAB script.

Supplementary Results

Design of sample collector

The manufacturing cost and dimensional accuracy of the parts manufactured with different printers were investigated. The material cost of the designs and time took to manufacture and process them are given in Fig. S3b. The material cost was calculated by using the material usage data supplied by the printer and up-to-date end-user prices. All three designs were printed with three different 3D printers (FormLabs, Makerbot and

Objet30) and shown in Fig S3a. Visually, the collectors printed by the Formlabs and Objet 30 have similar surfaces; on the other hand, the Makerbot-printed collector has a rough surface caused by higher layer thickness. Moreover, parts printed with Makerbot needed deburring and polishing with sandpaper to have smoother surface which took approximately 15 minutes per part. Due to the nature of inkjet-based printing, the Objet30 requires support material inside cavities. Support material needs to be cleaned after printing, and this took approximately 15 minutes for each part. The support material also increases surface roughness. FormLabs prints need to be cleaned from support connections with pliers, then soaked in an IPA bath for at least 20 minutes to remove uncured resin from the surface. After the IPA bath, the surface of the print was polished with sandpaper where the support connections were present, resulting in a total of 30 minutes post-processing time per part. The Makerbot prints cheaper and faster, which makes it better for prototyping. However, the post-processing requires more manual labor and the rougher surface is more prone to a build-up of sample, and thus cross-contamination. In addition, both Makerbot and Objet30 held less sample at lower angles, mostly due to the comparatively rougher surface. FormLabs was shown to be the better option in terms of consistency (less manual labor) and long-term use (smoother surface). The dimensional accuracy of the parts was tested by measuring the dimeter and length at multiple locations. The results in Fig. S3c show that all three printers are very accurate dimensionally. To calculate the volume of the parts, they are measured and documented density values were used.

Supplementary Figures

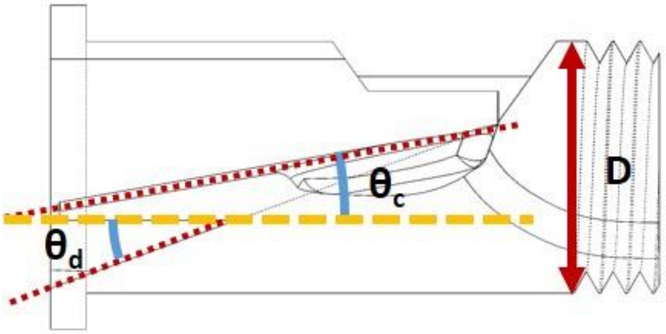

Figure S1. Schematic of the collector design parameters. The design diameter (*D*), collection angle (θ_c), and drain angle (θ_d), shown on a cross-sectional view of a representative sample collector.

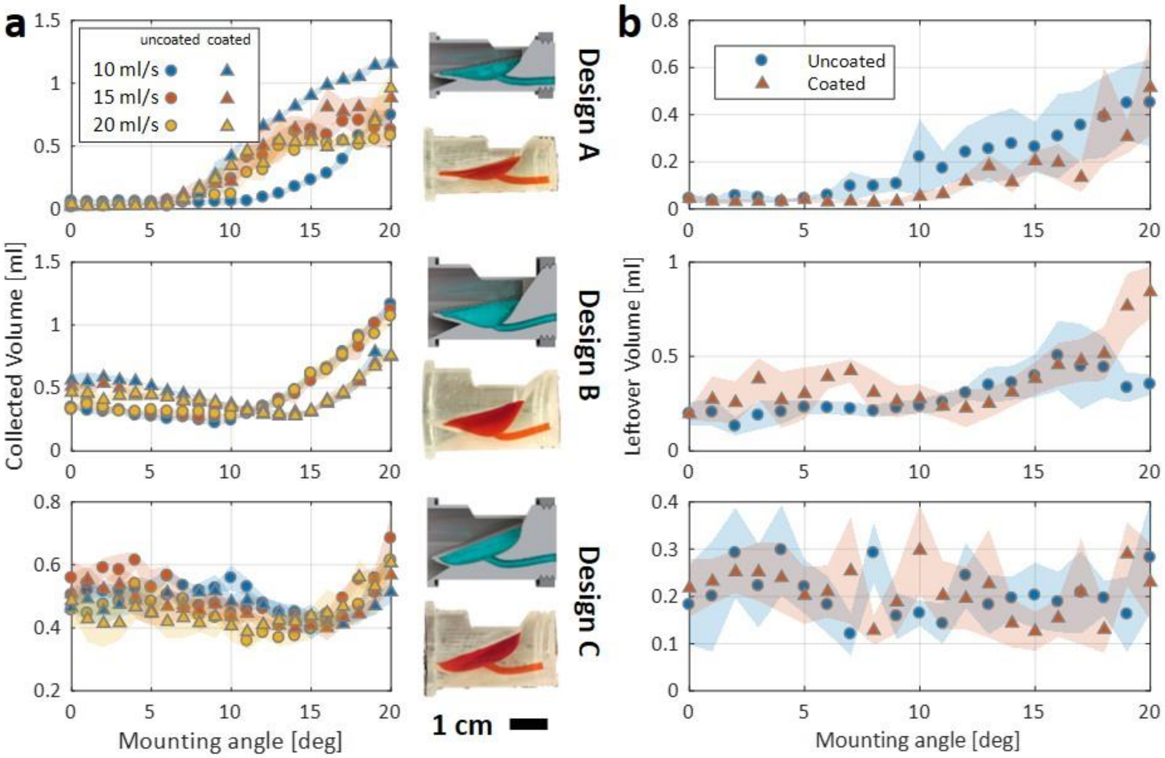

Figure S2. Characterization of the amount of sample collected and leftover sample volumes by various design iterations. (a) The amount of sample collected (mL) versus mounting angle (degrees) (n = 5) for each design iteration. The cross-section of the three design that have been characterized are given next to corresponding result (computer model, above; photograph, below). **(b)** The amount of leftover liquid after flushing for each design iteration.

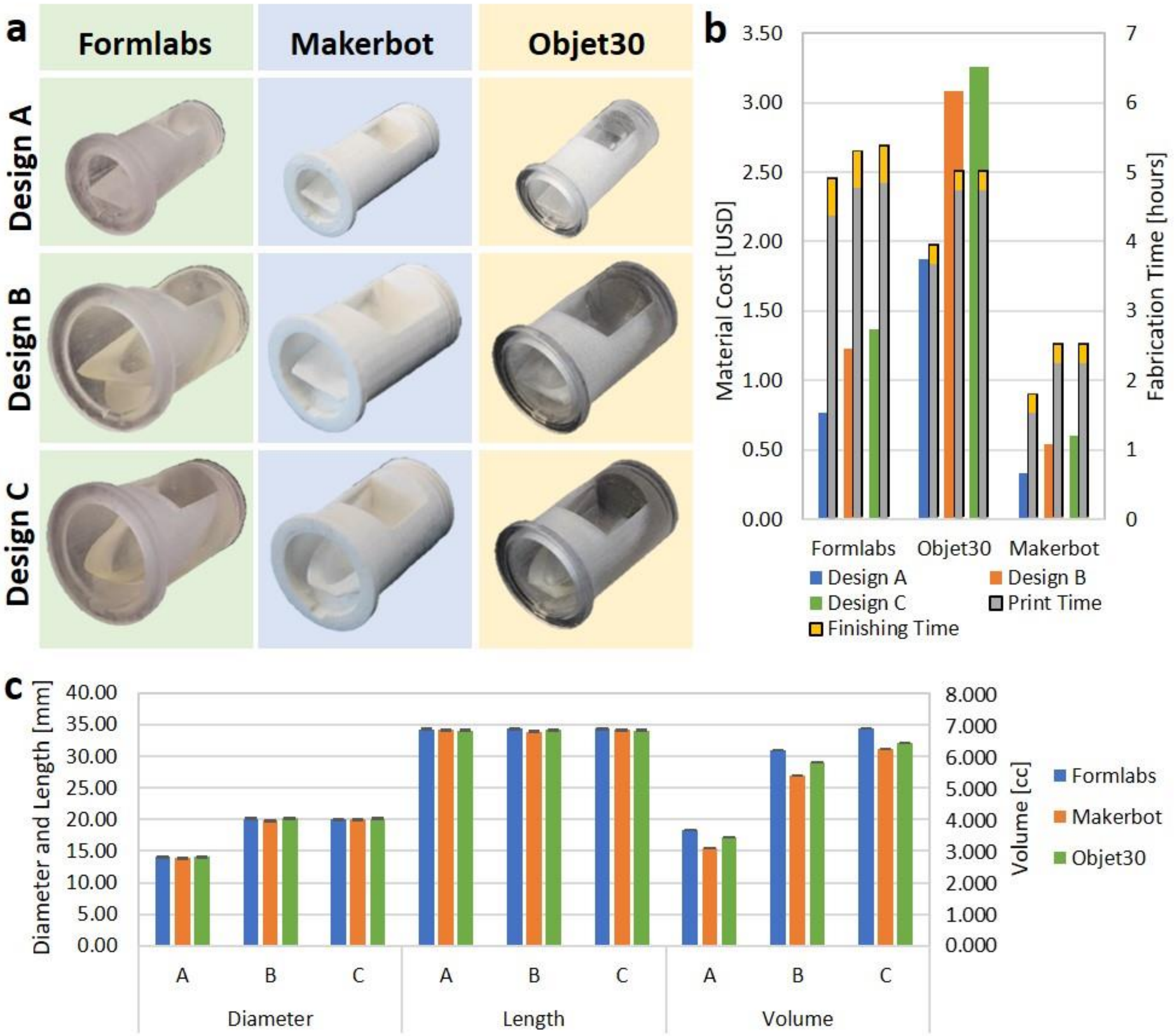

Figure S3. Comparison of 3D-printing performance for sample collector design iterations printed using Formlabs Form 2, Makerbot Replicator, and Objet30 Prime. (a) Images of printed prototypes. (b) Cost and time comparison of different printers and designs. Finishing time includes any post-treatment and deburring necessary to use the part. (c) Measured dimensions and volumes of the printed parts (error bars show standard deviation, but are too small for visibility).

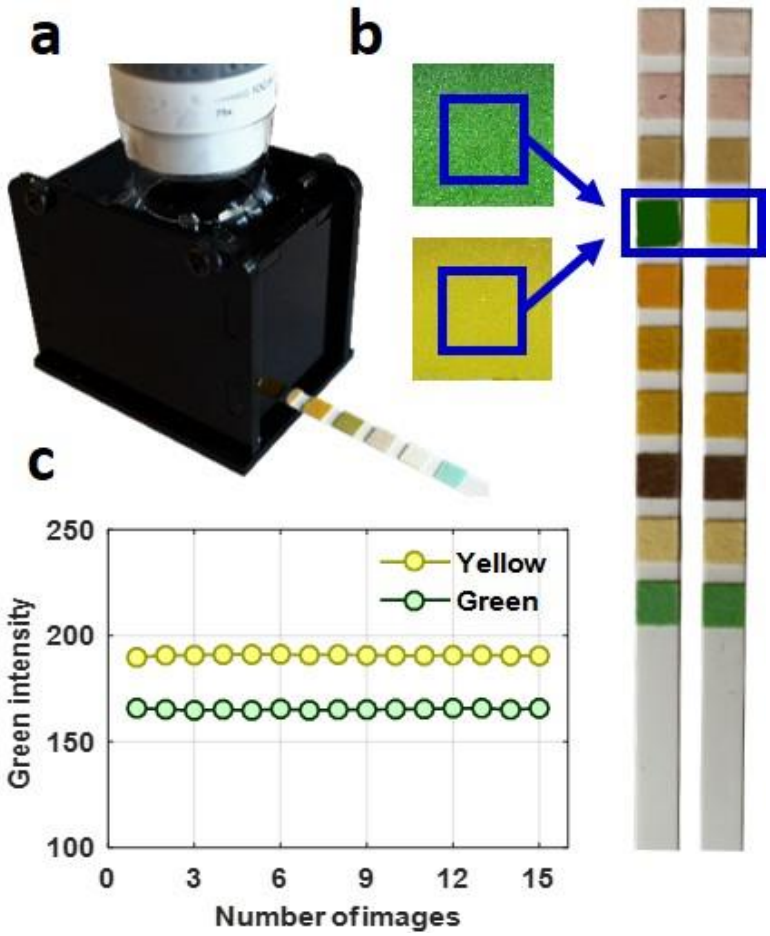

Figure S4. Validation of dipstick imaging. (a) Imaging box to block outside light to capture an image of an inserted dipstick. **(b)** Color samples affixed to dipsticks. **(c)** Repeated green intensity measurements of color samples show consistency in imaging.
